# Purification and analysis of endogenous human RNA exosome complexes

**DOI:** 10.1261/rna.057760.116

**Published:** 2016-09

**Authors:** Michal Domanski, Paula Upla, William J. Rice, Kelly R. Molloy, Natalia E. Ketaren, David L. Stokes, Torben Heick Jensen, Michael P. Rout, John LaCava

**Affiliations:** 1 Laboratory of Cellular and Structural Biology, The Rockefeller University, New York, New York 10065, USA; 2 Centre for mRNP Biogenesis and Metabolism, Department of Molecular Biology and Genetics, Aarhus University, 8000 Aarhus C, Denmark; 3 Skirball Institute and Department of Cell Biology, New York University School of Medicine, New York, New York 10016, USA; 4 Simons Electron Microscopy Center at New York Structural Biology Center, New York, New York 10027, USA; 5 Laboratory of Mass Spectrometry and Gaseous Ion Chemistry, The Rockefeller University, New York, New York 10065, USA; 6 Institute for Systems Genetics and Department of Biochemistry and Molecular Pharmacology, New York University School of Medicine, New York, New York 10016, USA

**Keywords:** RNA exosome, protein complex purification, ribonuclease

## Abstract

As a result of its importance in key RNA metabolic processes, the ribonucleolytic RNA exosome complex has been the focus of intense study for almost two decades. Research on exosome subunit assembly, cofactor and substrate interaction, enzymatic catalysis and structure have largely been conducted using complexes produced in the yeast *Saccharomyces cerevisiae* or in bacteria. Here, we examine different populations of endogenous exosomes from human embryonic kidney (HEK) 293 cells and test their enzymatic activity and structural integrity. We describe methods to prepare EXOSC10-containing, enzymatically active endogenous human exosomes at suitable yield and purity for in vitro biochemistry and negative stain transmission electron microscopy. This opens the door for assays designed to test the in vitro effects of putative cofactors on human exosome activity and will enable structural studies of preparations from endogenous sources.

## INTRODUCTION

The eukaryotic RNA exosome is a multisubunit complex harboring both exo- and endonuclease activities derived from enzyme components situated upon a catalytically inactive structural core that bears similarities to bacterial PNPase (for review, see Wasmuth and Lima 2012b). The exosome has been shown to act on a prodigious number of substrates, yet exhibit specificity in its activity, leading to a great deal of interest in the associated mechanisms. *Saccharomyces cerevisiae* has been the most attractive model organism from which to prepare endogenous exosomes owing to its tractable genetics and haploid growth, which allows for single-copy epitope-tagging of the endogenous gene(s) of interest. Moreover, yeast cultures are easily and cheaply grown at large-scale, facilitating the preparation of copious amounts of complex for purification and biochemical/structural analyses (e.g., Allmang et al. 1999; LaCava et al. 2005; Hernández et al. 2006; Dziembowski et al. 2007; Wang et al. 2007). Although the abundance of exosome complexes in human cells is not a limiting factor (estimated at ∼40,000 copies per cell in log-phase U2OS cell cultures [Beck et al. 2011]), preparing large enough quantities of cells expressing, but not overexpressing, tagged exosome complexes is relatively expensive and time consuming. An additional hurdle to studying human exosomes has been the lack of effective procedures to obtain abundant and pure complexes from the typically smaller quantity of starting material obtained from human tissue culture.

While common themes exist, details of exosome-mediated processing pathways markedly differ between yeast and human, and appear to be modulated by cell-compartment-specific core components and accessory factors (Lykke-Andersen et al. 2011; Sloan et al. 2012; Chlebowski et al. 2013), motivating us to study endogenous exosome complexes obtained from human cells. For example, affinity captured human exosomes readily copurify the DExH-box helicase SKIV2L2 (Mtr4p in yeast) (Chen et al. 2001; Lubas et al. 2011; Domanski et al. 2012), whereas yeast exosomes do not (Allmang et al. 1999; Synowsky et al. 2009)—despite evidence that SKIV2L2/Mtr4p is an exosome accessory factor in both organisms (for review, see Sloan et al. 2012). Additionally, a canonical ribonuclease component, DIS3 (Rrp44p in yeast), is relatively stable in yeast (Allmang et al. 1999; Dziembowski et al. 2007) but has proven averse to copurification with the human exosome (Chen et al. 2001; Staals et al. 2010; Tomecki et al. 2010). It has also been proposed that yeast and human exosome components may differ in their modes of substrate-level activity (Januszyk et al. 2011); and human exosome pathways may take advantage of a larger number of cofactors/adaptors (Lubas et al. 2011; Beaulieu et al. 2012; Andersen et al. 2013; Bresson et al. 2015). Hence, endogenous exosomes purified from human cells are valuable but elusive targets for biochemical, enzymatic, and structural study.

We previously addressed the technical limitations hindering highly efficient recovery of affinity tagged exosome complexes (Domanski et al. 2012); and recently, we explored and optimized the extraction procedure for the preparation of compositionally distinct exosome populations—including those exhibiting the retention of DIS3 within the complex (Hakhverdyan et al. 2015). Building on these methods, we have now developed additional protocols for the preparation of larger quantities of active endogenous human exosomes. We have examined our preparations structurally by negative-stain transmission electron microscopy (TEM) and explored their ribonucleolytic properties using in vitro assays.

## RESULTS

### Purifying endogenous human exosomes

**FIGURE 1.**
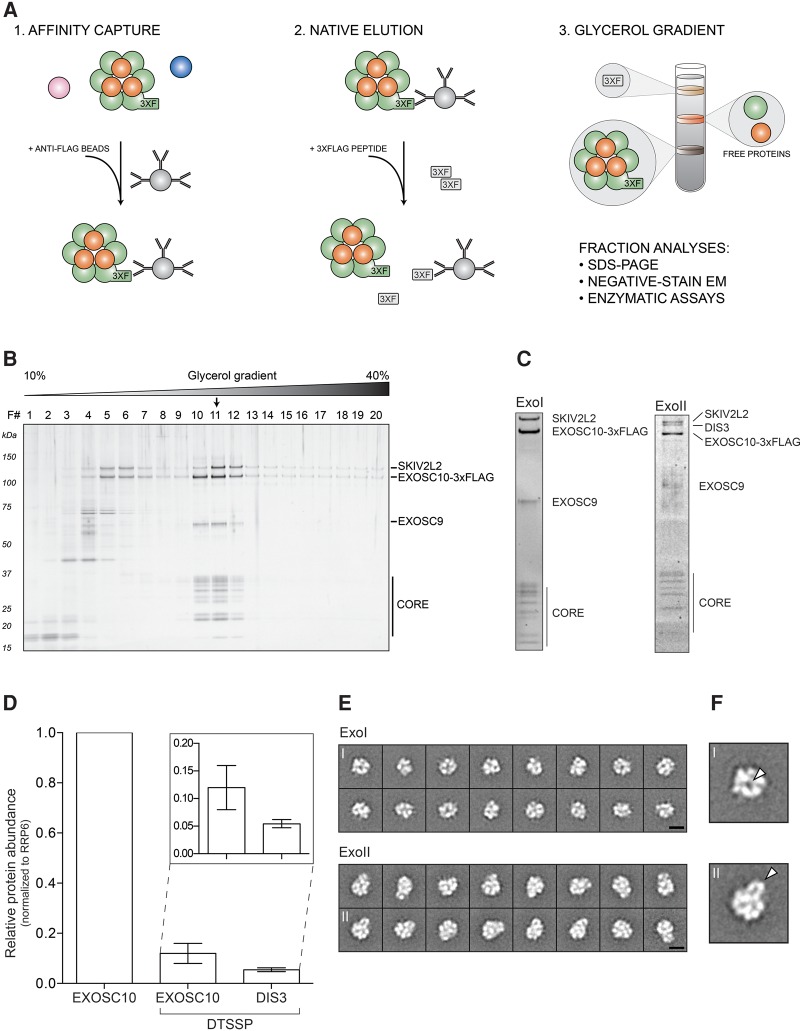
Purification and analysis of endogenous human exosomes. (*A*) (1) 3xFlag-tagged exosome component is affinity captured using anti-Flag antibodies coupled to magnetic beads. Pink and blue circles represent proteins not related to the exosome. (2) Native elution is performed with 3xFlag peptide. (3) Eluted complexes are further fractionated on a glycerol gradient and analyzed as indicated. (*B*) Silver stained SDS polyacrylamide gel displaying the fractionation of ExoI on a 10%–40% glycerol gradient. Exosome constituents are labeled; bands marked “core” consist of the low molecular mass components EXOSC1-8, MPHOSPH6, and C1D. Black arrow indicates the peak fraction. (*C*) Peak fractions from ExoI and ExoII glycerol gradients analyzed by SDS-PAGE. The ExoI peak fraction was stained with silver, ExoII with Sypro Ruby. Protein bands are labeled as in *B*. (*D*) MS-based estimate of the relative amounts of EXOSC10 and DIS3 obtained in velocity sedimented fractions of ExoI and ExoII preparations, as in *C*. Error bars indicate the data range. (*E*) Negative-stain TEM analysis of ExoI and ExoII particles. Shown are 16 representative 2D class averages for each preparation. Scale bars (black): 10 nm. (*F*) ExoI and ExoII 2D class averages have been enlarged to illustrate the “hole,” observed in ExoI class averages and the “lobe,” observed in ExoII class averages (white arrows).

To produce endogenous human RNA exosomes at large-scale, we conditioned normally adherent HEK-293 Flp-In T-REx cells expressing tetracycline inducible 3xFlag-tagged EXOSC10 (human RRP6) to suspension growth (method adapted from Taylor et al. 2013), which yielded ∼10 g wet cell weight (WCW) per 400 mL of culture medium. We utilized protocols incorporating scaled-up versions of our previously described methods (Domanski et al. 2012; Hakhverdyan et al. 2015), in conjunction with glycerol density gradient rate zonal centrifugation (diagrammed in Fig. 1A; a representative gradient shown in Fig. 1B), to produce distinct exosome populations differentiated by the absence (ExoI) or presence (ExoII) of the component DIS3 (Fig. 1C). All the canonical exosome components were detected in both preparations by mass spectrometry: EXOSC1-10, SKIV2L2, MPHOSPH6, and C1D (data not shown). While we were able to obtain a homogenous peak fraction of ExoI (Fig. 1B, fraction 11), gradient purification of ExoII proved more challenging because DIS3 dissociated from the exosome during centrifugation. To mitigate this issue, we tested the ability of the homobifunctional, amine-reactive and reversible crosslinking reagent DTSSP to stabilize our ExoII preparations. The successful use of DTSSP permitted us to gradient purify fractions of ExoII (Fig. 1C, right panel). Note, when the ExoI and DTSSP crosslinked ExoII are sedimented using identical gradient conditions (see Materials and Methods, SW 55 Ti rotor), the ExoII peak runs approximately one-half of a fraction (∼1 mm) further into the gradient compared to ExoI. Using label-free quantitative mass spectrometry (method modified from Andersen et al. 2013, see Materials and Methods) we estimated the yield of EXOSC10 to be approximately 10 times higher in the ExoI preparation than in the ExoII prep (Fig. 1D), commensurate with the much higher total exosome yield of that protocol as judged by protein staining (data not shown). DIS3 was not detectably cofractionated with ExoI during the purification, whereas we estimate DIS3 to be approximately one-half as abundant as EXOSC10 in ExoII preparations (Fig. 1D).

### Analysis by TEM

Given the compositional differences, we examined the ExoI and ExoII preparations by negative-stain TEM to see whether we could identify the structural differences. The selected particles were subjected to iterative stable alignment and clustering (ISAC) that extracts validated, homogeneous subsets of images (Yang et al. 2012). Analysis of ExoI by ISAC resulted in 46 class averages accounting for 4905 particles (74% of the entire data set) and analysis of ExoII resulted in 55 class averages accounting for 5096 particles (72% of the entire data set). Both preparations included some class averages exhibiting aspects of the characteristic doughnut-like shape also observed in other studies using reconstituted or endogenous exosomes from *S. cerevisiae* (Malet et al. 2010; Liu et al. 2014, 2016) and *L. tarentolae* (Cristodero et al. 2008). However, a notable difference in the overall shape between the ExoI and ExoII preparations was also observed (Fig. 1E). While ExoI particles were smaller and more ring-shaped, exhibiting a “hole” presumably corresponding to the central channel of the exosome ring, the ExoII particle contained an extra lobe of density that partially obscured the appearance of the central pore (Fig. 1F).

### Testing RNase activity

**FIGURE 2.**
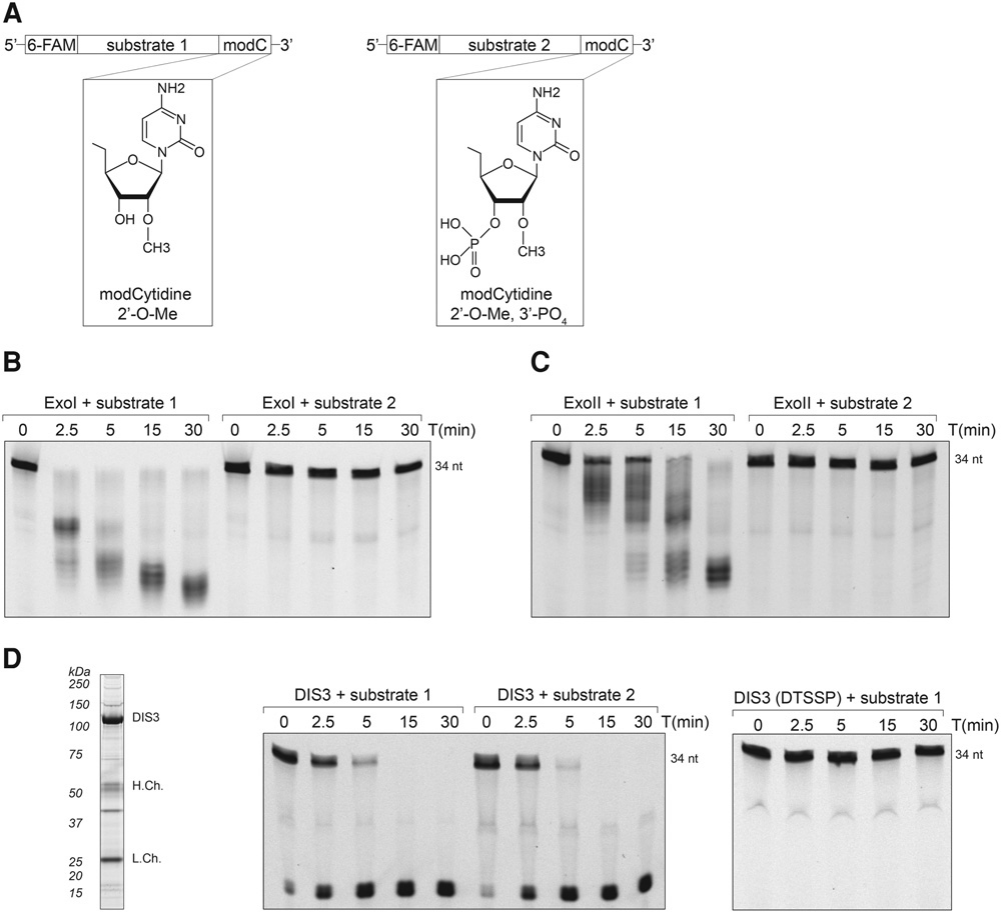
Preparations of endogenous human exosomes exhibit specific 3′-to-5′ distributive, exoribonucleolytic activity. (*A*) Structure comparison of 3′-end modified oligos utilized for RNA degradation assays. Substrate 1 is 2′-*O*-methylated, while substrate 2 is also 3′-phosphorylated. Both substrates are labeled with 6-FAM (6-carboxyfluorescein) at the 5′-end. (*B*) RNA degradation intermediates resolved by denaturing urea-polyacrylamide gel electrophoresis; ExoI was incubated with either substrate 1 or 2 for the indicated time. (*C*) RNA degradation intermediates produced by ExoII, incubated with RNA substrates as in *B*. (*D*) Panel of assays testing the effect of DTSSP on DIS3 enzymatic activity. (*Left*) Coomassie stained SDS-PAGE displaying DIS3-3xFlag purification from HEK293 cells (H.Ch. and L.Ch.: IgG heavy and light chains, respectively). (*Center*) DIS3 incubated with both RNA substrates, as in *B*. (*Right*) DTSSP-treated DIS3 was incubated with substrate 1 and the RNA degradation products were separated as in *B*.

We next examined the in vitro RNase activity of the ExoI and ExoII preparations. In order to perform RNA degradation assays, it was essential to concentrate the gradient purified exosomes by recapturing them upon α-Flag affinity medium. Once immobilized, the exosome preparations were challenged with generic, 5′-fluorescein amidite (6-FAM) labeled RNA substrates: substrate 1 containing a free 3′-OH group and substrate 2 containing a 3′-phosphate group, as diagrammed in Figure 2A. The latter modification serves as a control in which hydrolytic 3′→5′ exoribonuclease activity is inhibited by preventing the terminal residue from being properly oriented in the active site (Cudny et al. 1981; Beese and Steitz 1991; Burkard and Butler 2000).

When incubated with substrate 1, ExoI exhibited a distributive degradation pattern comparable to that previously shown to be characteristic of hydrolytic 3′→5′ exoribonuclease activity as exhibited by heterologously expressed EXOSC10 (Burkard and Butler 2000; Januszyk et al. 2011; Wasmuth and Lima 2012a); we also observed that this activity was blocked by a 3′-phosphate (Fig. 2B), further supporting this directionality and mechanism. Comparable results were obtained with our preparation of ExoII (Fig. 2C), supporting an EXOSC10-based activity only (Wasmuth and Lima 2012a) and hence inactivation of DIS3. Note, the lower level of distributive EXOSC10-derived activity is consistent with the 10-fold-reduction of EXOSC10 of the ExoII preparation (Fig. 1D).

To determine whether DTSSP treatment might have affected the activity of DIS3 within the exosome, we tested the activity of DIS3-3xFlag purified from HEK-293 cells, before and after treatment with DTSSP. Figure 2D displays the results obtained. RNA degradation assays using DIS3-3xFlag purified from HEK-293 cells exhibited processive character and this degradation was insensitive to 3′-PO_4_ (Fig. 2D, middle panel), consistent with previous observations (Tomecki et al. 2010) and DIS3's endonucleolytic ability. However, after treatment with DTSSP, DIS3 was inactive (Fig. 2D, right panel), suggesting that the procedure for stabilizing the DIS3/exosome complex in the ExoII fraction has negative effects on DIS3 enzymatic activity.

**FIGURE 3.**
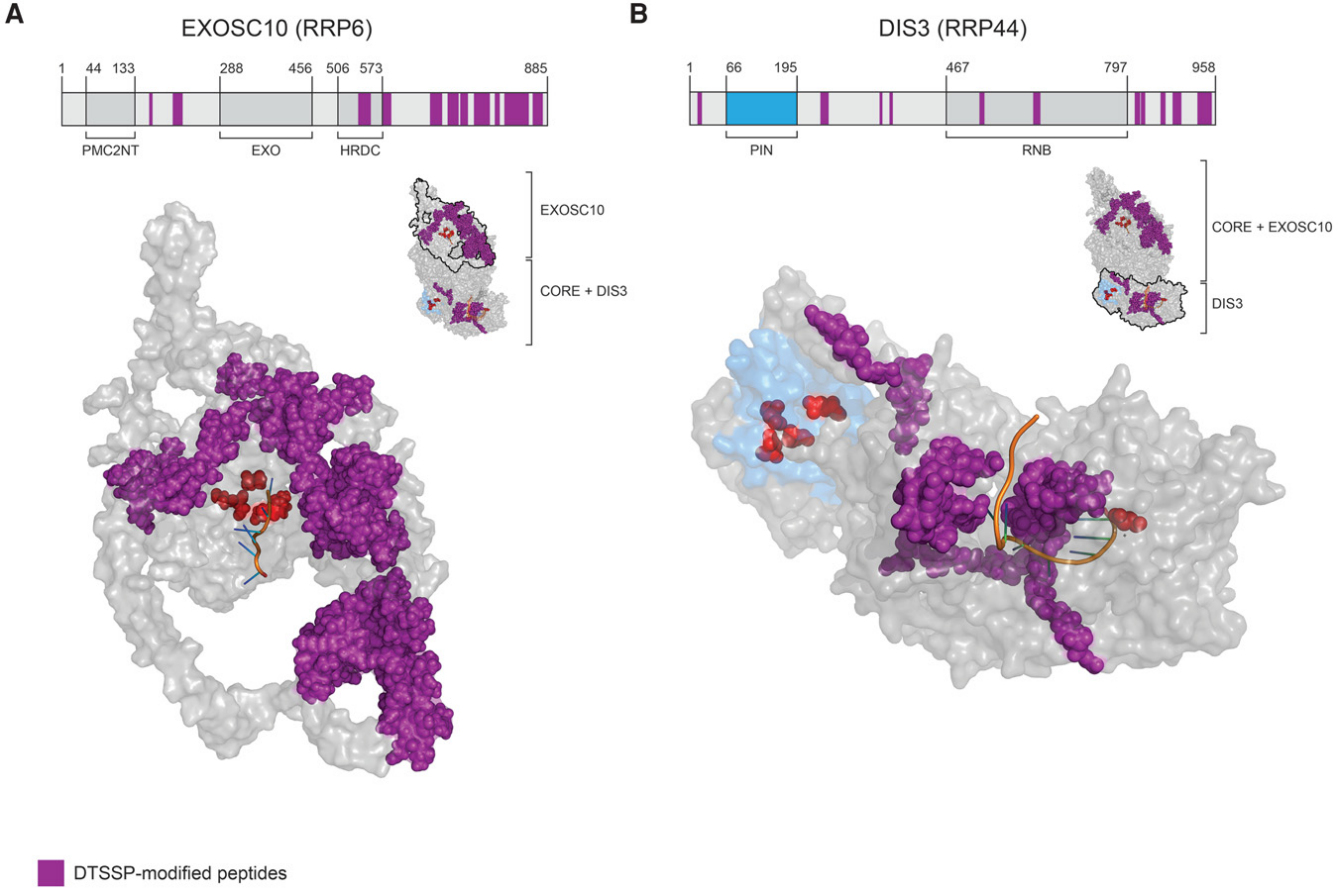
DTSSP-modified peptides mapped to EXOSC10 and DIS3 protein sequences and structures. (*A*, *upper*) Linear arrangement of EXOSC10 domains with DTSSP-modified peptides mapped (in purple). The domain organization is based on the protein sequences obtained from the uniprot.org and pfam.xfam.org databases. (*Lower*) The in silico modeled structure of EXOSC10 using *I-TASSER* (Yang and Zhang 2015; Yang et al. 2015). Highlighted are DTSSP-modified peptides (purple), key active site residues (red), and cocrystalized RNA (orange). The protein orientation is indicated by the miniature structure of a 12-component exosome (PDB ID: 5c0w with EXOSC10 model), *upper right*. (*B, upper*) Arrangement of DIS3 domains represented as in *A*. (*Lower*) DTSSP-modified peptides were first mapped onto the human DIS3 protein sequence and the equivalent residues were then mapped on the yeast DIS3 structure (PDB ID: 5c0w), displayed, and colored as in *A*. Additionally, the PIN domain was labeled in blue to distinguish it from the RNB domain. The protein orientation is indicated as in *A*.

In an attempt to rationalize the inactivation of DIS3 by the crosslinking treatment, while EXOSC10 remained relatively unaffected, we mapped the locations in EXOSC10 and DIS3 of DTSSP-modified peptides detected by tandem mass spectrometry; these are displayed in Figure 3 and listed in Table 1. We noted that several modified peptides could be found within the RNA-binding path, proximal to the exonucleolytic active site in DIS3 (Fig. 3B); whereas key RNA binding residues in EXOSC10 were not observed to be modified by DTSSP (Fig. 3A). The PIN-domain of DIS3, containing the endonucleolytic active site, was not observed to be modified by DTSSP. Notably, distinct conformations of DIS3 have been characterized by multiple structural biology approaches (Januszyk and Lima 2014; Makino et al. 2015; Shi et al. 2015; Liu et al. 2016), leading to speculation that DIS3 may exhibit structural dynamics important for its activity; such dynamics may be stifled by crosslinking (see Discussion).

**TABLE 1.**
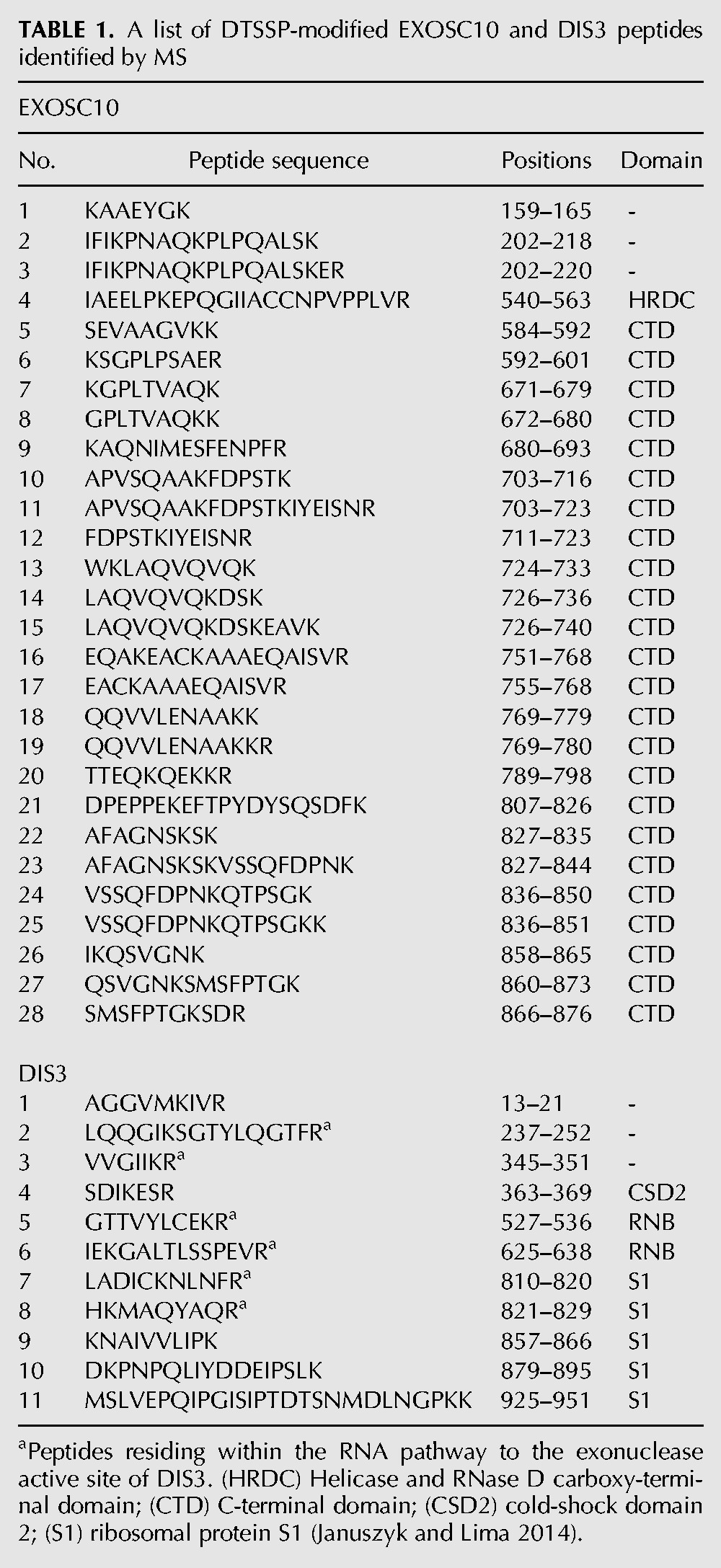
A list of DTSSP-modified EXOSC10 and DIS3 peptides identified by MS

## DISCUSSION

Key among our results, the purified exosome preparations described exhibited a distributive exoribonucleolytic activity commonly associated with EXOSC10-containing exosomes (Wasmuth and Lima 2012a) and sufficient yield and purity were achieved to enable EM-based structural studies. Thus, the presented methodology should enable a range of assays on human exosomes authentically expressed and assembled in vivo. We believe that these will prove to be of high value for in vitro reconstitutions of endogenous human exosomes with putative cofactor complexes (e.g., Lubas et al. 2011; Andersen et al. 2013), analogous to what has been done previously in yeast (e.g., LaCava et al. 2005; Vanacova et al. 2005), as well as to explore specific RNA substrate-level effects on human exosome activity.

Although the association of DIS3 within the human exosome has proven labile once released from the cell (Chen et al. 2001; Staals et al. 2010; Tomecki et al. 2010), we recently discovered conditions that enabled its effective copurification (Hakhverdyan et al. 2015). Nevertheless, during subsequent glycerol gradient centrifugation, DIS3 ultimately dissociated from exosomes. To counteract dissociation, we used DTSSP to retain DIS3 with the exosome. This treatment stabilized the association of DIS3 with the exosome through gradient centrifugation, and we carried out negative stain TEM imaging on gradient fractionated exosomes in the absence (ExoI) and presence (ExoII) of DIS3. The analyses revealed that the ExoI and ExoII preparations described were relatively homogenous and sufficiently concentrated, yielding ∼50 2D class averages comprising ∼5000 particles (>70% of total) in both cases. These metrics indicate that the particle sets would likely serve as good material for future cryo-EM studies that may permit accurate modeling of the 3D structures. Unfortunately the DTSSP treatment compromised DIS3 enzymatic activity. Several DTSSP-modified peptides on DIS3 were located within regions critical for RNA binding. This may, at least in part, explain the observed inactivating effect of the treatment. Multiple conformational states of DIS3 have been characterized. An interaction between the PIN domain and a cold-shock domain, CSD2, was described as participating in a conformation important for channeling RNA to the exonucleolytic active site (Makino et al. 2013; Januszyk and Lima 2014). We observe that a peptide within the CDS2 domain was modified by DTSSP treatment (Table 1, DIS3 #4). Additionally, an N-terminal region within DIS3 has been shown to become structured upon RNA binding; we observed a DTTSP-modified peptide within this region (Table 1, DIS3 #1) (Makino et al. 2013, 2015).

In future work, we will explore alternative crosslinking strategies that may be able to recapitulate the desired stabilization without compromising DIS3 enzymatic activity. Being that DTSSP is a homobifunctional amine-reactive crosslinker, it may be necessary to explore alternative chemistries in order to avoid producing a similar effect. EDC, which links carboxyl groups with amino groups, has been used effectively as a complementary approach to amine-amine linkages (Shi et al. 2014). Formaldehyde is an alternative that exhibits diverse reactivity as well as reversibility (Sutherland et al. 2008)—providing for easy monitoring of reaction progress and gradient fraction composition by standard SDS-PAGE, comparable to what has been done in the present study.

Distinctive macromolecular complexes and alternative configurations of related complexes can be obtained intact from the endogenous source through rigorous affinity capture optimization (Hakhverdyan et al. 2015). Using endogenous human exosomes as a proof of concept, we have demonstrated that the purification procedures discovered are scalable—and this can be done cost-effectively by suspension conditioning normally adherent HEK-293 cells—providing excellent starting materials for further biochemical, enzymological, and structural study. Because this approach has significant advantages over in vitro assembly of components heterologously expressed in bacteria, which suffers from innate difficulties in expressing large proteins, the need to coexpress multiple interaction partners, and the lack of endogenous post-translational modifications (Fakruddin et al. 2013), we believe it will prove to be of great general utility in the study of macromolecular assemblies.

## MATERIALS AND METHODS

### Exosome purification

HEK-293 cells expressing EXOSC10-3xFlag were grown on dishes and cryogenically disrupted as previously described (Domanski et al. 2012) or grown in suspension (Muller et al. 2005; Taylor et al. 2013) with identical results. Affinity capture was performed utilizing a total of 1 g WCW of cell powder, prepared as four 250 mg purifications in parallel, combined before gradient centrifugation (described below). For protein extraction two solutions were used: (ExoI) 20 mM HEPES-Na pH 7.4, 300 mM NaCl, 1% v/v Triton X-100; (ExoII) 20 mM HEPES-Na pH 7.4, 100 mM NaCl, 5 mM CHAPS. Captured proteins were eluted using 3xFlag peptide (1 mg/mL) and subsequently fractionated by rate zonal centrifugation on 10%–40% v/v glycerol gradients. To obtain the ExoII gradient fraction, the 3xFlag peptide eluate of DIS3-containing exosomes was first treated with DTSSP before running the gradient, as described below. Gradients were produced using The Gradient Master (BioComp Instruments Inc.) and centrifuged using either an MLS-50 rotor in an Optima MAX Ultracentrifuge or an SW 55 Ti rotor in an Optima L Ultracentrifuge (Beckman Coulter Inc.) at 4°C using minimum acceleration and no brake. Fractions were automatically collected in 2 mm increments with a Piston Gradient Fractionator (BioComp Instruments Inc.) and analyzed on 4%–12% Bis–Tris SDS-polyacrylamide gels in MOPS buffer (as per manufacturer's instructions, Life Technologies) followed by silver (Life Technologies) or Sypro Ruby (Sigma-Aldrich) staining. Peak fractions used for RNase assays and negative-stain TEM (as descried below) were prepared as follows: ExoI was obtained from ∼fraction 11 after centrifugation at 50 k RPM for 8 h (MLS-50 rotor); ExoII was obtained from ∼fraction 6 after centrifugation at 40 k RPM for 8 h, or after centrifugation at 40 k RPM for 7 h on a 20%–50% v/v gradient (MLS-50 rotor). For quantitative MS (described below), equivalent fractions were obtained for ExoI and ExoII using an SW 55 Ti rotor (6 h 36 min): the ExoI peak sediments to fraction ∼11 and the ExoII peak sediments between fractions ∼11 and 12.

### MS-based quantitation of EXOSC10 and DIS3 levels in gradient purified fractions

ExoI was prepared in triplicate and ExoII in duplicate. For each replicate, 200 µL aliquots were taken from each of three fractions encompassing the sample peak sedimented in a glycerol gradient; these were methanol/chloroform precipitated (Wessel and Flügge 1984). The precipitated proteins from each aliquot were resuspended in 1× LDS (Life Technologies) and pooled, respectively, for each replicate. The pooled samples were run ∼4–6 mm into a 4%–12% Bis–Tris SDS-polyacrylamide gel, resulting in a “gel-plug” that was excised and processed by in-gel digestion (Shevchenko et al. 2006; LaCava et al. 2015). The gel-extracted peptides were gradient-eluted from a reverse phase C18 column and electrosprayed online into an Orbitrap Fusion (Thermo Fisher Scientific). Within each 4-sec cycle, ions were detected in a full scan in the Orbitrap, and fragmented by CID in decreasing intensity order. To account for and identify DSTTP-modified peptides in ExoII preparations, the spectra were searched by X!Tandem against the Ensembl human GRCh37 assembly with lysine and protein N-termini as potential sites of the CAMthiopropanoyl modification (+145.01975 Da; the product of DTSSP crosslink reversal). Intensity values were obtained from MaxQuant version 1.5.2.8 (Cox and Mann 2008) and used for label-free quantitation (Andersen et al. 2013). Briefly, the relative abundance of the proteins was calculated based on the peptide intensities obtained from the triplicate experiment for nontreated samples, and the duplicate experiment where DTSSP crosslinking was applied. In the first step, the average intensities were normalized to the molecular weight (MW) of each protein. Subsequently, calculated numbers were divided by the average intensity/MW value of the EXOSC10 from nontreated samples. The error bars represent the highest and the lowest values calculated for each protein. The plot was prepared using the GraphPad Prism software.

### DIS3 purification

HEK293 cells expressing DIS3-3xFlag were grown and cryomilled as above. The SDS-PAGE gel lane shown in Figure 2D (left panel) was obtained by denaturing elution (in 1× LDS) of DIS3-3xFlag from 200 mg of cell powder; extracted in buffer consisting of 300 mM NaCl, 20 mM HEPES pH7.4, 0.5% v/v Triton X-100, supplemented with protease inhibitors; using 20 µL slurry of affinity medium. For RNase assays (Fig. 2D, middle and right panel) DIS3 was affinity captured from 0.5 g of cell powder (2 × 250 mg) extracted as above, followed by native elution. After incubation with Dynabeads (2 × 25 µL slurry), beads were pooled, washed three times with 1 mL of extraction buffer, and once with 1 mL of washing buffer (100 mM NaCl, 20 mM Tris pH 8, 0.01% Triton X-100). After elution with 30 µL of 3xFlag peptide (1 mg/mL) the beads were washed with 30 µL washing buffer and both solutions were combined and tested for RNA degradation activity (see below).

### DTSSP crosslinking

Two microliters of a freshly prepared 1.2 mM DTSSP (Thermo Fisher Scientific) solution was added to 10 µL of DIS3+ exosomes (ExoII) prepared by elution with 3xFlag peptide as described above. Crosslinking was carried for 50 min at RT and the reaction was quenched by the addition of 2 µL 1 M Tris–Cl pH 8. To reverse crosslinks before the analysis by SDS-PAGE and protein staining, 25 mM of DTT was added and the samples were incubated at 75**°**C for 20 min.

### Mapping of crosslinks to DIS3 and EXOSC10 structures

CAM-thiopropanoyl modified peptides were identified during MS analyses (as described above) of ExoII preparations (with and without glycerol gradient fractionation). A cutoff filter of less than or equal to −2 was applied to the log(e) values of peptide-spectrum matches obtained from X!Tandem (Fenyö and Beavis 2003). The remaining peptides were mapped to DIS3 and EXOSC10 sequences and then onto either a 3D yeast structure (DIS3 → Rp44p), or in the case of EXOSC10, a 3D model of the human protein. Full details are as follows: For DIS3, human (Q9Y2L1) and yeast (Q08162) DIS3 sequences were aligned using Clustal omega (Sievers et al. 2011; McWilliam et al. 2013; Li et al. 2015). DTSSP-modified peptides were mapped on the human DIS3 and the equivalent residues were then mapped on the yeast DIS3 (Rrp44p) structure (PDB ID: 5c0w). For EXOSC10, the 3D structure of EXOSC10 (the human ortholog of the yeast Rrp6 protein) was generated using the modeling program I-TASSER (Yang and Zhang 2015; Yang et al. 2015). The final model was generating using the structures of yeast Rrp6p (5c0w [chain K], 5c0w [chain A], 5c0x [chain K]), and EXOSC10 (3saf [chain A]) as the top threading templates. I-TASSER assesses the quality of the model using a C-score. The C-score is a value describing overall model quality that ranges from 2 to −5. The final model resulted in a C-score of −2.26 reflecting reasonable confidence in the model generated. The model of EXOSC10 was aligned with the structure of yeast Rrp6p (5c0w) with an rmsd of 0.336. The DTSSP-modified peptides were mapped on the model of EXOSC10. All structures presented were visualized using the program PyMol v1.7.4 (Schrödinger, LLC).

### TEM

A 3 µL drop of gradient-purified ExoI or ExoII fractions were applied to glow-discharged carbon-coated copper grids. These were stained with three drops of 1% uranyl formate and air-dried. Images were collected on a JEOL JEM-2100F transmission electron microscope (JEOL USA Inc.) operating at 200 keV at a magnification of 50,000× and 1.5 µm underfocus. Images were recorded on a TVIPS F224HD 2048 × 2048 CCD camera with 24 µm pixels (Tietz Video and Image Processing Systems GmbH). The pixel size at the specimen level was 2.93 Å. Particles were selected using Boxer from EMAN (Ludtke et al. 1999) and normalized. The contrast transfer function (CTF) of the images was determined using ctfit from EMAN and the phases flipped accordingly. The particles were centered and then subjected to the iterative stable alignment and clustering (ISAC) (Yang et al. 2012) technique to produce stable class averages. The program was run for five generations to classify ExoI and eight generations to classify ExoII. After each generation stable particles were removed from the stack and the program was re-run with unclassified particles until no new classes were found. A pixel error of 2√3 was used for the stability threshold.

### RNA degradation assays

Gradient purified exosomes (three peak fractions) were affinity immobilized on 10 µL of α-Flag magnetic medium for 30 min at 4**°**C with mixing and washed once with 1 mL of a solution comprised of 20 mM Tris–Cl pH 8, 100 mM NaCl, 0.01% v/v Triton X-100. The medium was then resuspended in 25 µL of the same solution. The RNase activity of affinity immobilized exosomes was then assayed under conditions comparable to those previously described (Greimann and Lima 2008; Januszyk et al. 2011): To the 25 µL suspension described above, 25 µL of a 2× reaction buffer consisting of 20 mM Tris–Cl pH 8, 20 mM DTT, 0.5 mM MgCl_2_, 5% v/v RNasin (Promega, #N2515), and 0.4 pmol/µL RNA oligo (described below), were added—yielding a 50 µL reaction volume. The mixture was then incubated at 37°C with gentle mixing (1000 RPM in Thermomixer; Eppendorf). Aliquots (10 µL) were taken at different time points and mixed 1:1 with 2× RNA loading buffer (95% formamide, 20 mM EDTA, 1% v/v DNA loading dye; Thermo Fisher Scientific, #R0611), heated for 30 sec at 80°C and run at 15 W on a 20% denaturing urea-polyacrylamide gel in TBE buffer (National Diagnostics, #EC-829). DIS3 RNase activity was tested essentially as for gradient purified exosomes. However, DIS3-3xFlag was not immobilized on the beads; instead 25 µL of a native eluate (see DIS3 purification) was mixed directly with 25 µL of 2× reaction buffer.

Fluorescence imaging to monitor reaction progress was carried out on a Fujifilm LAS-3000. The RNA oligo substrate used was an HPLC-purified, 34-mer with the sequence: 5′-CCUAUUCUAUAGUGUCACCUAAAUGCUAGAGCUC-3′ (a 5′-truncated form of the generic substrate presented in Januszyk et al. 2011) synthesized with a 5′ 6-FAM (fluorescein) group to enable fluorescence detection and a 2′-*O*-methylation to enable uniform 3′-phosphorylation of blocked substrates (Integrated DNA Technologies).
